# Effects of sulforaphane on breast cancer based on metabolome and microbiome

**DOI:** 10.1002/fsn3.3168

**Published:** 2023-03-31

**Authors:** Shuyuan Cao, Shengjie Hu, Ping Jiang, Zhan Zhang, Lei Li, Qian Wu

**Affiliations:** ^1^ Center for Global Health, School of Public Health and Department of Health Inspection and Quarantine School of Public Health, Nanjing Medical University Nanjing China

**Keywords:** breast cancer, metabolism, microbiome, one‐carbon metabolism, sulforaphane

## Abstract

Sulforaphane (SFN) is a promising phytochemical with a wide range of antitumor activities. A comprehensive understanding of the effects of SFN on breast cancer based on the metabolome and microbiome is limited. Thus, we treated MCF‐7 cell‐transplanted nude mice with 50 mg/kg SFN. SFN inhibits breast cancer cell proliferation. SFN increased the levels of sulfate‐related metabolites and glutathione‐related metabolites and decreased tryptophan metabolites and methyl‐purine metabolites in urinary metabolic profile. SFN indirectly affected the activation of aryl hydrocarbon receptor by tryptophan metabolism. The ratio of SAM to methionine was decreased by SFN while the global DNA methylation was downregulated in tumor tissue. SFN decreased the sulfate‐reducing bacterium *Desulfovibrio*, which is related to reduced methylation capacity, and increased the genus *Lactobacillus* related to tryptophan metabolites with antitumor activities. In conclusion, we provide a perspective on the metabolome and microbiome to elucidate the antitumor activities of SFN.

## Graphical abstract

This paper provided promising insights into the role of SFN on breast cancer based on metabolome and microbiome. SFN influenced one‐carbon metabolism, especially the ratio of SAM to Methionine, by which SFN can regulate DNA methylation and gene expression. SFN altered the diversity of gut microbiota to indirectly affect the methylation activity and antitumor activities by AHR signaling pathway.

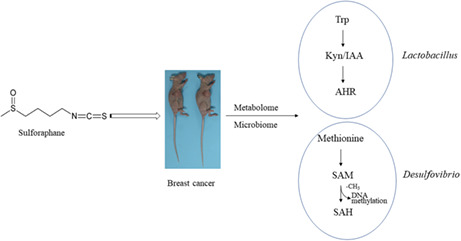

## 1 INTRODUCTION

Breast cancer has become the most common malignant tumor in women in the world (Grogan Fleege & Cobain, 2022). The well‐known pathogenesis of breast cancer involves two mechanisms. One is mediated by estrogen receptor‐induced transcriptional activation of target genes, and the other is involved in oxidative metabolism of estrogens (Starek‐Swiechowicz et al., 2021). Recently, many studies have shown that dietary factors play a key role in reducing the risk of breast cancer (Ghazi et al., 2020; Ngo & Williams, 2021).

Sulforaphane (SFN) is a promising anticancer phytochemical which is widely distributed in the Brassicaceae family, such as cabbage, broccoli, and brussels sprouts (Nandini et al., 2020; Ruhee & Suzuki, 2020). SFN has been demonstrated to inhibit carcinogenesis through various mechanisms including antioxidant, anti‐inflammatory, immunomodulatory, and antiproliferation effects (Kaiser et al., 2021; Wu et al., 2020). Our previous study has revealed that SFN may exert anticell proliferation effects by altering purine metabolism and amino acid metabolism and antioxidative effects by altering glutathione metabolism (Cao et al., 2018). These results indicated that SFN can influence biological pathway by metabolism. SFN may also act as a direct or indirect epigenetic modulator of gene transcriptional activity (Dos Santos et al., 2020; Mitsiogianni et al., 2020). Although some studies support that sulforaphane is involved in epigenetic changes, there is a limited number of research in illustrating the mechanism of sulforaphane to modulate the epigenome, especially in breast cancer. DNA methylation is mediated by DNA methyltransferases (DNMT) that use S‐adenosylmethionine (SAM) as a methyl donor (Hudlikar et al., 2021; Paul et al., 2018). SAM, which is synthesized from one‐carbon metabolism, serves as the common methyl donor in mammals. One‐carbon metabolism, including the folate and methionine cycles, supports glutathione (GSH) and SAM synthesis and provides methyl groups for DNA methylation and histone methylation as well (Newman & Maddocks, 2017). Therefore, the abundance or availability of SAM can directly affect the methylation status of DNA. Methylation reactions involve a methyl group transfer from SAM to cytosine residues of DNA and generate S‐adenosylhomocysteine (SAH) as by‐products that can be redirected into the methionine cycle. Thus, the ratio between SAM and methionine, and SAM and SAH is an important reflection of the methylation status. DNA methylation and one‐carbon metabolism are highly connected, which might help further understand the effect of SFN on breast cancer.

In addition, disturbance of gut microbiota has been shown to play a role in the development of breast cancer (Ruo et al., 2021). The gut microbiota plays an essential role in regulating the development and maintenance of the host immune system (Wastyk et al., 2021). Some studies have demonstrated that these commensal communities have impacts on the efficacy of antitumor therapy through the modulation of host immunity (Huang et al., 2020; Wang et al., 2014). What's more, these metabolites derived from differential gut microbiota, in turn, have effects on host disease, which may include tryptophan metabolites mediating aryl hydrocarbon receptor (AHR) activation (Thakur & Chen, 2019).

On the basis of the relevance of metabolome and microbiome to the effect of SFN and the importance of gut commensal bacteria in host immune system maintenance, in this present study, we constructed a murine breast cancer model to make a comprehensive investigation into alterations of metabolome and microbiome under the interventions of SFN. There will provide a better understanding of SFN effects on breast cancer.

## 2 MATERIALS AND METHODS

### 2.1 Establishment of murine breast cancer model

Five‐week‐old female BALB/c‐nude mice were obtained from Shanghai Slac laboratory animal co., Ltd. The method of establishment of murine breast cancer model was modified according to our previous work (Wu et al., 2012). After 1‐week acclimatization, the mice were injected with MCF‐7 breast cancer cells (5 × 10^7^/ml) (gifted by Prof. Yuhue SUN, Nanjing Medical University). The volume of tumors was calculated by the following formula: (diameter × radius^2^)/2. When tumors reached a size of 100 mm^3^, the murine breast cancer model was successfully established. Then, these mice were equally and randomly divided into model group and SFN‐treated group. The SFN‐treated group was intragastrically administered with SFN (LKT Laboratories, Inc.) at a dose of 50 mg/kg twice a week while the model group received water correspondingly. Urine and feces were collected twice a week throughout the experimental period. After 6 weeks of administration, the mice were humanely sacrificed. Tumor tissues were fixed with 4% paraformaldehyde and embedded in paraffin wax or frozen at −80°C for further detection.

### 2.2 Immunohistochemical analysis

For immunohistochemical analysis, the paraffin‐embedded sections of tumor tissues were deparaffinized and rehydrated. Antigen retrieval was conducted and endogenous peroxidase activity was blocked. Then, the sections were incubated with Ki67 antibodies and the following HRP‐labeled secondary antibody. DAB chromogenic reaction was conducted and the sections were counterstained with nucleus. After being dehydrated and mounted, stained slides were scanned by Pannoramic SCAN (3DHISTECH Kft) and were quantified and analyzed by Halo v3.0.311.314 software. The Ki‐67 expression levels were expressed as the percentage of cells with positive nuclear staining among the total number of tumor cells. Detection of apoptosis was conducted by the TUNEL assay kit (Servicebio). Confocal analysis (Nikon Eclipse C1 and Nikon DS‐U3) of paraffin‐embedded breast tumor was visualized by staining with FITC (green) and DAPI (blue). The green cells indicated the TUNEL‐positive apoptotic cells.

### 2.3 Metabolic profiles by UPLC‐Orbitrap‐MS

Preparation of all samples was performed on ice. Urine samples were thawed and diluted at a ratio of 1:3 with methanol (v/v) to remove the large molecular weight proteins. The rest part was centrifuged at 11,000 *g* for 15 min and the supernatants were transferred to a vial and stored at 4°C until further analysis. Metabolic profiling was performed on an ultra‐performance liquid chromatography (UPLC) Ultimate 3000 system (Dionex), coupled with an Orbitrap mass spectrometer (Thermo Fisher Scientific). The chromatographic separation was performed on a Hypersil Gold C18 column (100 mm × 2.1 mm, 1.9 μm, Thermo Fisher Scientific) and set the temperature at 40°C. A multistep gradient consisting of 0.1% formic acid in water (A) and methanol (B) was applied. The flow rate was 0.4 ml/min, which was achieved by linearly increasing the concentration of solvent B from 5% to 95% in 15 min, and then sustained with 95% solvent B for 2 min before being re‐equilibrated in 5% solvent B. The UPLC autosampler temperature was set at 4°C and the injection volume was 5 μl. The Q Exactive ion source settings in both positive and negative modes were as follows: the spray voltage of 3.5 kV, the capillary temperature of 300°C, and the flow of the auxiliary gas, sweep gas, and S‐Lens RF level was 10, 2, and 50 arbitrary units, respectively. In the full scan, the mass resolution was set at 7 × 10^5^ with an automatic gain control target of 1 × 10^6^ charges and a maximum injection time of 120 ms. The quality control (QC) samples had been prepared by pooling same volume of urine from all samples and analyzed interval to ensure stability and repeatability. Meanwhile, the mass spectrometer was calibrated every 24 h to ensure mass accuracy.

The data were processed as we described before (Huang et al., 2020). The raw data were converted to get the primary database by MS convert software. The database was imported into the XCMS online metabolomics (The Scripps Research Institute) afterward to derive and preprocess including peak realignment, baseline correction, and peak deconvolution. The filter conditions were conducted and then the multivariate statistics, such as principal component analysis and partial least squares discriminant analysis (PLS‐DA), were performed using SIMCA‐P 14.0 software (Umetrics). After the data were classified and dimensionality reduced, the candidate variables were selected by variable importance in the projection (VIP) larger than 1.0 and *p* value < .05. Those variables were considered statistically significant and were aligned and identified according to their m/z, retention time, and HMDB Library (http://www.hmdb.ca/) in the following analysis.

### 2.4 Targeted analysis of tryptophan metabolites

The methods of tryptophan metabolites detection were described in detail in our previous work (Wu et al., 2022). In brief, L‐Tryptophan (Trp), Kynurenic acid (Kyn), 2‐picolinic acid (PIC), quinolinic acid (QUI), indole acrylic acid (IA), indole‐3‐propionic acid (IPA), and tryptamine from Aladdin Biochemical Technology Co., Ltd; 5‐hydroxyindoleacetic acid (5‐HIAA), indole acetaldehyde (IAAld), indoleacetic acid (IAA), and 5‐hydroxytryptamine, (5‐HT) hydrochloride from Sigma Aldrich; 5‐hydroxy‐L tryptophan (5‐HTP) from MAYA‐Reagent; Indole‐3‐aldehyde (IAld) from TCI (Shanghai) Development Co., Ltd., were determined in this study. Tryptophan‐d5 (Trp‐d5) (Toronto Research Chemicals) was used as internal standards. A quantity of 50 μl of urine was added to 150 μl of methanol. The internal standard solution was spiked at 50 ng/ml concentration. The supernatant was quantified using UPLC Ultimate 3000 system (Dionex) with an Orbitrap mass spectrometer (Thermo Fisher Scientific). The separation of the samples was performed on a Hypersile C18 column (100 mm × 2.1 mm, 1.9 μm) at a flow rate of 0.3 ml/min. Mobile phase A was water containing 0.1% (v/v) formic acid, and mobile phase B was acetonitrile containing 0.1% (v/v) formic acid. The column temperature was at 40°C. The injection volume of samples was 10 μl. Mass spectrometric analyses in the positive ion mode in full scan mode and the parameters are given in Table S1. The temperature of the turbo ion electrospray was set at 300°C. The ion spray voltage was 3500 V.

### 2.5 Targeted analysis of one carbon metabolites

Samples were pretreated according to a previous study (Wang et al., 2014). For metabolite quantitation, labeled one‐carbon metabolites including SAH‐d4 and Met‐d4 from Toronto Research Chemicals were used as internal standards. Briefly, the tissue homogenate was added with 1 ml of methanol containing 100 μg/ml ascorbic acid, 100 μg/ml citric acid, and 1.5 mg/ml DTT. The internal standard solution was spiked at 200 ng/ml concentration. After vortexed for 2 min and centrifuged at 17000 *g* for 15 min at 4°C, the supernatant was dried under nitrogen at room temperature. The residue was reconstituted with 100 μl of methanol/water (3:1, v/v) containing 10 μg/ml of ascorbic acid, citric acid, and DTT, and stored at −20°C for further analysis. The calibration curve standards (5‐MT, Ser, Gly, Met, SAM, SAH, Hcy, and Betaine from Sigma Aldrich) were prepared by spiking the internal standard solutions. Metabolites were analyzed by UPLC Ultimate 3000 system (Dionex) with an Orbitrap mass spectrometer (Thermo Fisher Scientific). The separation of the samples was performed on a Waters ACQUITY BEH‐C18 column (2.1 mm × 100 mm, 1.7 μm) at a flow rate of 0.3 ml/min. Mobile phase A was water containing 20 mM ammonium formate and 0.15% (v/v) formic acid, and mobile phase B was methanol containing 0.15% (v/v) formic acid. The column temperature was at 35 ± 1°C. The injection volume of samples was 20 μl. The effluent was unsplitted. Mass spectrometric analyses in the positive ion mode in full scan mode and the parameters are given in Table S2. The temperature of the turbo ion electrospray was set at 320°C. The ion spray voltage was 3200 V.

### 2.6 Bisulfite sequencing PCR

DNA was extracted from tumor tissue using TIANamp Genomic DNA Kit (TIANGEN Biotech Co., Ltd.) according to the instructions from the manufacturer. DNA methylation of LINE1 gene was assessed by bisulfite sequencing PCR (BSP). For *LINE‐1*, BSPCR primer sequences were 5′‐TTATTAGGGAGTGTTAGATAGTGGG‐3′ for forward; 5′‐CCTCTAAACCAAATATAAAATATAATCTC‐3′ for reverse. Genomic DNA was used for bisulfite treatment using the EZDNA Methylation™ Kit (Zymo Research). The bisulfite‐treated DNA was amplified with methylation‐specific primers using GoTaq Green Master Mix (Promega). PCR products were purified by Gel Extraction Kit (E.Z.N.A.) and subcloned into pMD 19‐T Vector (TaKaRa). Ten clones from each sample were sequenced (TsingKe Biological Technology). Sequencing data were analyzed with the DNAMAN to examine the methylation status. The percentage of DNA methylation was calculated by the following the formula: (methylated CG/CG) × 100%.

### 2.7 Quantitative reverse transcription PCR (RT‐qPCR)

Total RNA was extracted from frozen tissue using TRIzol reagent (Invitrogen). Then, RNA was transcribed into cDNA by PrimeScript (Takara). Quantitative PCR amplification was performed using TB Green (Takara). The primers for *DNMT1* gene: forward, 5′‐GATCGAGACCACGGTTCCTC‐3′; reverse, 5′‐CGGCCTCGTCATAACTCTCC‐3′. The primers for *AHR* gene (Licznerska et al., 2021): forward, 5′‐ACAGATGAGGAAGGAACA GAG‐3′; reverse, 5′‐CTT GCT TAG AGT GGA TGTGG‐3′. *GAPDH* was as internal control.

### 2.8 Microbial diversity by 16S rRNA gene sequencing

Microbial DNA from stool samples was extracted by E.Z.N.A.® soil kits (Omega Bio‐tek) following the manufacturer's instructions. The V3‐V4 variable region of 16S rRNA gene was amplified by universal primers (338F: 5′‐ACTCCTACGGGAGGCAGCAG‐3′; 806R: 5′‐GGACTACHVGGGTWTCTAAT‐3′) (ABI GeneAmp® 9700). PCR products were purified with an AxyPrep DNA Gel Extraction Kit (Axygen Biosciences). The DNA library was constructed using the TruSeq TM DNA Sample Prep Kit. Sequencing was performed on the MiSeq PE300 (Illumina) platform. The raw sequences were assembled by the Fast Length Adjustment of Short reads software after quality filtering by Trimmomatic software. Chimeras were removed by UCHIME. Operational taxonomic units (OTUs) were generated with 97% similarity using UPARSE (version 7.1 http://drive5.com/uparse/). OTUs were illustrated according to the RDP classifier (http://rdp.cme.msu.edu/), mapped with Silva database (SSU128), with a confidence threshold of 70%. A diversity analysis was performed. The significant differences between the groups were determined by Wilcoxon rank‐sum test corrected with the FDR. All data were analyzed on the free online Major‐bio I‐Sanger Cloud Platform (www.i‐sanger.com).

### 2.9 Statistical analysis

The differences in cell proliferation, gene expression, and metabolites were determined by Student's *t*‐test by GraphPad Prism 8 software (GraphPad Software). Differences were considered statistically significant at *p* < .05.

## 3 RESULTS AND DISCUSSION

### 3.1 SFN inhibited cell proliferation in breast cancer

**FIGURE 1:**
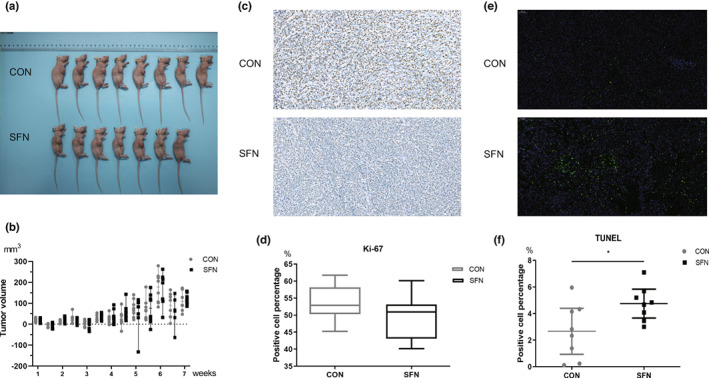
The effects of SFN on the growth of breast cancer in female BALB/c‐nude mice. (a) The view of breast tumor in mice. *n* = 8 (control), *n* = 7 (SFN). (b) The growth of breast tumor during experimental period. (c, d) Comparison of the percentage of Ki67‐positive cells between two groups. (e, f) Comparison of the percentage of TUNEL‐positive cells between two groups. Data represented as the mean ± SD. *p < .05, compared to the control.

Compared with the control group, no significant change in the tumor volume was observed in SFN group (Figure 1a,b). There was a slight decrease of ki‐67 but no statistical significance after SFN intervention (Figure 1c,d). SFN group showed a significant elevated level in apoptosis compared with the control group (Figure 1e,f). Although the tumor size had no reduction, it still indicated that SFN may induce apoptosis to inhibit cell proliferation in breast cancer.

### 3.2 SFN changed metabolic profile in breast cancer

**FIGURE 2:**
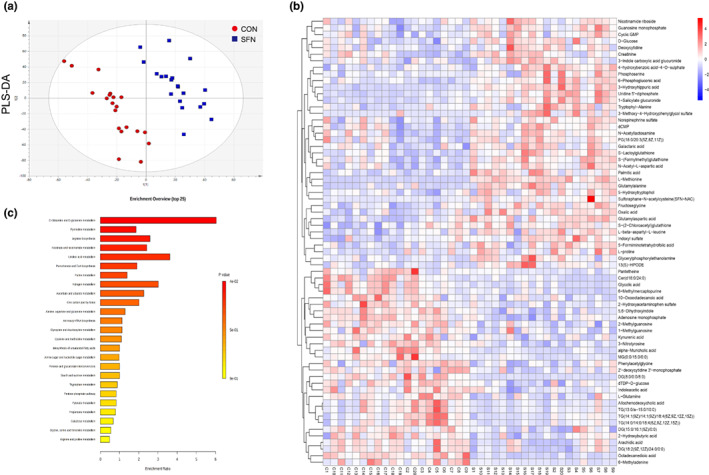
The effects of SFN on the metabolic profiles of breast cancer in female BALB/c‐nude mice. (a) PLS‐DA score plots showed metabolic pattern between the control and SFN groups. (b) Heatmap of differential metabolites between the control and SFN groups. Red color represents an upregulation of the metabolite while the blue color represents a downregulation. (c) Pathway enrichment of differential metabolites after SFN intervention.

The urinary metabolic profile was obtained according to the chromatographic and mass spectrum conditions described above. To investigate the differences between groups, multivariate analysis was performed. The PLS‐DA score plots (Figure 2a) showed a clear separation which indicated the different metabolic patterns between the control and SFN‐treated groups. The variable importance for projection (VIP) was obtained from the PLS‐DA along with the *q* value and was applied to select the different metabolites (VIP value >1.0 and *q* value <0.05). We found that 68 metabolites were significantly changed after SFN intervention. The differential metabolites are listed in Table S3. The peak intensity of these metabolites was plotted for a heat map (Figure 2b). The map displayed a more intuitive difference and showed that N‐acetylcysteine sulforaphane, a metabolite of SFN, was significantly increased in SFN group. Moreover, the sulfate‐related metabolites, such as indoxyl sulfate and 4‐hydroxybenzoic acid‐4‐O‐sulphate, were increased in the SFN group. It suggested that the level of sulfurization in vivo was elevated by SFN intervention. It has been reported that many thiol‐containing compounds, such as SFN and isothiocyanate, display antioxidant activity to balance the aberrant oxidative stress in pathology (Zhu et al., 2021). With lipophilicity and nucleophilicity, SFN can activate Nrf2 pathway, exhibiting cytoprotective effects by reduction of oxidative damage in cells (Yang, Zahid, et al., 2013). Meanwhile, high levels of glutathione‐related metabolites, S‐lactoylglutathione, and S‐(formylmethyl)glutathione were detected in SFN group which was associated with the antioxidant effect of SFN. Pathway analysis revealed that d‐glutamine and d‐glutamate metabolism, pyrimidine metabolism, linolenic acid metabolism, and purine metabolism were mainly enriched (Figure 2c). In addition, kynurenic acid and indoleacetic acid were decreased in SFN group, which indicated that the pathway of tryptophan (Trp) metabolism was disturbed. Methylated purine metabolites, such as 1‐methylguanosine, 2‐methylguanosine, and 6‐methyladenine, decreased significantly after SFN intervention, which can act as methylation marker, and we also noticed that levels of some one‐carbon metabolites, such as SAM and methionine were elevated in SFN group.

### 3.3 SFN indirectly affected the activation of AHR by tryptophan metabolism

**FIGURE 3:**
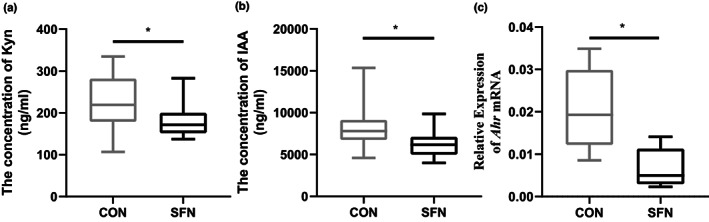
The effects of SFN on tryptophan metabolism and AHR expression. (a, b) The concentration of differential tryptophan metabolites in urine. (c) The expression of AHR in tumor tissue. Data represented as the mean ± SD. *p < .05, compared to the control.

We further performed targeted analysis of tryptophan metabolic pathway in urine of mice. Tryptophan metabolism includes three main pathways: (1) Intestinal microbiota directly converts tryptophan into indole and other derivatives (Zelante et al., 2013); (2) The kynurenine Pathway (KP) in intestinal epithelial cells is mediated by indoleamine 2, 3‐dioxygenase (IDO1) (Clarke et al., 2013); (3) 5‐hydroxyl tryptamine (5‐HT) in intestinal chromaffin cells is produced through tryptophan hydroxylase 1 (TpH1) pathway (Yano et al., 2015). The results of targeted detection of tryptophan metabolites showed that intestinal microbial metabolites, such as kynurenic acid and indoleacetic acid, were significantly decreased in SFN group, which can act as AHR ligands (Figure 3). The decreasing of AHR ligands may reduce the activation of AHR and may consequently reduce the expression of phase I metabolic enzymes such as CYP1A1 and CYP1B1, which can inhibit the production of carcinogenic intermediate metabolites, such as DNA adducts (Licznerska et al., 2015; Yang, Zhuang, et al., 2013). Reports have demonstrated that AHR was overexpressed in human breast tumors (Mohamed et al., 2019; Romagnolo et al., 2015), which was related to cell cycle progression (Barhoover et al., 2010) and immune tolerance (Ehrlich et al., 2016). Our study showed that SFN indirectly affected the expression of AHR by tryptophan metabolism, which indicated that SFN shows great promise as oncogenic treatment via the AHR pathway.

### 3.4 SFN altered one‐carbon metabolite pattern and DNA methylation status

**FIGURE 4:**
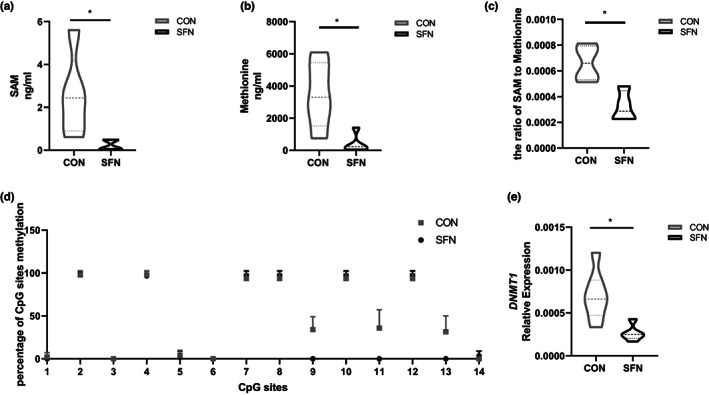
The effects of SFN on one‐carbon metabolism and DNA methylation. (a) SAM. (b) Methionine. (c) The ratio of SAM to methionine. (d) The DNA methylation percentage of CpG sites in the promoter of *LINE1* gene. (e) The expression level of *DNMT1* gene. Data represented as the mean ± SD. *p < .05, compared to the control.

We further performed a targeted analysis of one‐carbon metabolites. One‐carbon metabolism mediated by folic acid cycling is used to activate and transfer one‐carbon units for biosynthesis (Ducker & Rabinowitz, 2017). Tetrahydrogen folic acid (THF) accepts one‐carbon units derived from serine, yielding 5,10me‐THF. 5,10me‐THF is demethylated by methyltransferase to produce THF which continues to participate in the folic acid cycle. Homocysteine can recycle the methyl group removed from 5,10 me‐THF back to methionine (Tibbetts & Appling, 2010). Methionine receives adenosine produced by ATP breakdown to form S‐Adenosine methionine (SAM). After SAM methyl is transferred to receptors such as DNA, SAM becomes S‐adenosine homocysteine (SAH), which is then converted to homocysteine and completes the methionine cycle (Newman & Maddocks, 2017). The results showed that the levels of core one‐carbon metabolites, SAM, and methionine in SFN group were decreased (Figure 4a,b). Meanwhile, the ratio of SAM to methionine was observed to be reduced compared with the control group. Decreased ratio suggested attenuated methylation ability in SFN group (Figure 4c). Besides, we analyzed the CpG sites of the *LINE‐1* gene in tumor tissue by BSP method and the result indicated that SFN decreased the methylation level of the part of CG sites (Figure 4d), the methylation status of which can be a surrogate marker for genome‐wide methylation status (Lisanti et al., 2013). In addition, we also detected the expression of *DNMTs* in tumor tissues and found that the expression of the *DNMT1* gene was downregulated in SFN group. Our results were consistent with our previous work, which demonstrated that sulforaphane was able to reverse the estrogen‐induced DNA methylation silenced of the Catechol‐O‐methyltransferase (COMT) gene (Wu et al., 2019). It suggested that SFN altered the one‐carbon metabolite pattern and DNA methylation status.

### 3.5 SFN influenced microbial diversity in breast cancer

**FIGURE 5:**
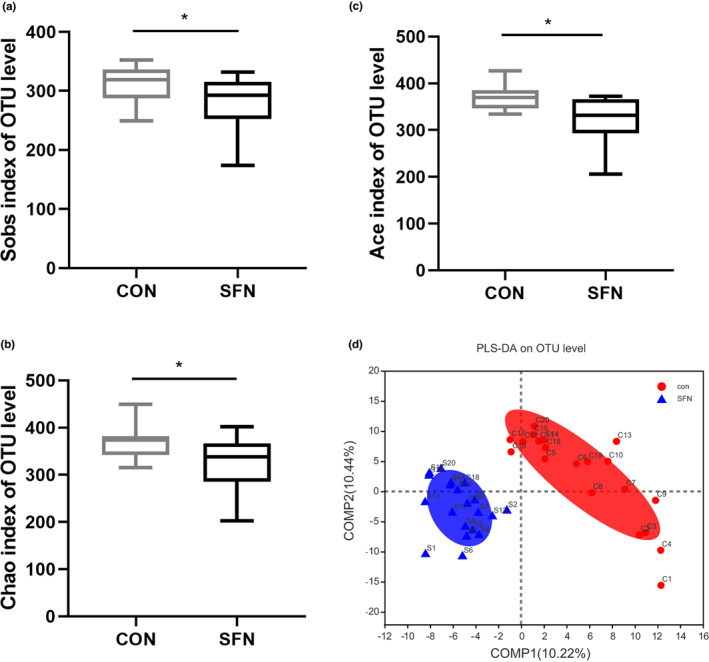
The effects of SFN on microbial diversity in breast cancer mice. (a–c) The alpha diversity analysis between groups was estimated by the Sobs, Chao, and Ace indices. (d) PLS‐DA plot showed gut microbiota metabolic profiles between the control and SFN groups. Data represented as the mean ± SD. *p < .05, compared to the control.

**FIGURE 6:**
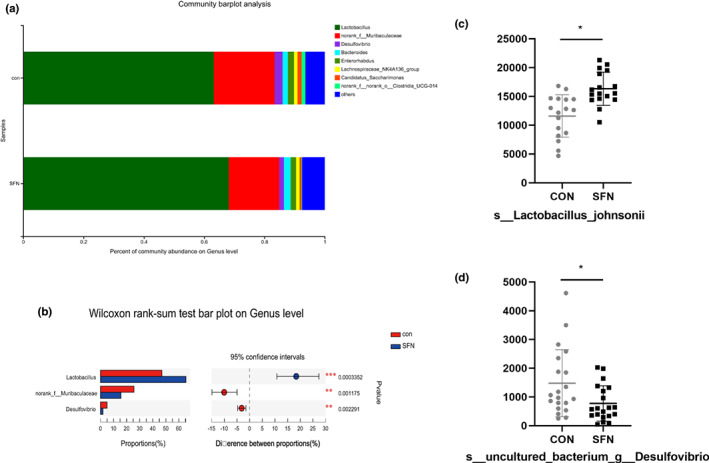
The effects of SFN on microbial community in breast cancer mice. (a) Bar plot of community abundance at the genus level. (b) Difference between the proportions of the gut microbiota at the genus level. Differences were analyzed by the Wilcoxon rank‐sum test. (c) Difference in s‐*Lactobacillus johnsonii* between groups. (d) Difference in s‐*Desulfovibrio* between groups. Data represented as the mean ± SD. *p < .05, compared to the control.

16S rRNA gene sequencing was performed to obtain the diversity of fecal microbiota. After sequence optimization and database annotation, the differences of OTU composition were observed after SFN intervention. The community richness of fecal microbiota at the OTU level was decreased in three different indexes, including Sobs, Ace, and Chao (Figure 5a–c). And PLS‐DA plots displayed a clear discrimination between the two groups (Figure 5d). The changes in the fecal microbiota between the groups were explored using community bar plot analysis and Wilcoxon rank‐sum test. Genus composition abundance map showed no differences between the groups (Figure 6a). *Desulfovibrio*, an anaerobic sulfate‐reducing bacterium, was significantly less abundant in the fecal microbiota of SFN group compared with the control group (Figure 6b,d), while *Lactobacillus*, especially *Lactobacillus johnsonii* was significantly more abundant in the fecal microbiota of SFN group (Figure 6b,c). It was reported that glucoraphanin (including sulforaphane) decreased the relative abundance of the family Desulfovibrionaceae, which is known as potential endotoxin producer (Nagata et al., 2017). The genus *Desulfovibrio* is capable of producing methyl‐mercury, but the mechanism of methylation by this microorganism remains unknown (Brown et al., 2011). Research in children with autism indicated that this microorganism may have reduced methylation capacity (James et al., 2004). Another study reported that intake of sulforaphane can enrich the abundance of the genus *Lactobacillus* (Wei et al., 2022). In a study by Behzadi et al. (2021), the MCF‐7 human breast cancer cells were treated with culture supernatants of *Lactobacillus acidophilus*. It exhibited antitumor activities in vitro and in vivo. What's more, *Lactobacillus* has the capacity to produce AHR ligands, including tryptophan metabolites (Valladares et al., 2013; Zhao et al., 2021). So, SFN may ameliorate breast cancer by AHR signaling pathway through microbial tryptophan metabolites.

## 4 CONCLUSION

Sulforaphane acting as a phytochemical agent has a wide range of anticancer activities. This paper provided promising insights into the role of SFN on breast cancer based on metabolome and microbiome. The results of this study indicated that SFN influenced one‐carbon metabolism, especially the ratio of SAM to methionine, by which SFN can regulate DNA methylation and gene expression. What's more, SFN altered the diversity of gut microbiota to indirectly affect the methylation activity and antitumor activities by AHR signaling pathway.

## FUNDING INFORMATION

This work was supported by the National Natural Science Foundation of China (81903365 and 82073630); Natural Science Foundation of Jiangsu Province (BK20161571), Natural Science Foundation of the Higher Education Institution of Jiangsu Province (16KJA330002). The funders had no role in the study design, data collection and analysis, decision to publish, or preparation of the manuscript.

## ACKNOWLEDGEMENTS

Authors thank Animal Core Facility Nanjing Medical University for their support.

## CONFLICT OF INTEREST

The authors declare that they do not have any conflict of interest.

## ETHICAL APPROVAL

The present experiment was performed in compliance with the Guidelines for the Care and Use of Laboratory Animals of Nanjing Medical University and approved by the Animal Ethical and Welfare Committee of Nanjing Medical University (No. 2007025).

## Supporting information


Table S1

Table S2

Table S3

## Data Availability

The data that support the findings of this study are available from the corresponding author upon reasonable request.
