# Supplementary material for: Effects of sulforaphane on breast cancer based on metabolome and microbiome
Source: Food Sci Nutr. 2023 Mar 31;11(5):2277–87. doi: 10.1002/fsn3.3168 (PMC10171519; doi:10.1002/fsn3.3168)
Supplement: Supplementary file 1 — Table S1 [file FSN3-11-2277-s003.docx]

**supplementary Table 1 MS instrumental parameters for tryptophan metabolites**

| Metabolites | m/z | RT | Regression equation | R^2^ |
| --- | --- | --- | --- | --- |
| PIC | 124.0393 | 0.76 | Y = -0.1797+0.015858*X | 0.9957 |
| QUI | 168.0291 | 1.03 | Y = -0.101052+0.00248201*X | 0.9946 |
| 5-HTP | 221.0920 | 1.77 | Y = -0.0975199+0.00283419*X | 0.9994 |
| 5-HT | 177.1019 | 2.11 | Y = -0.039639+0.00334171*X | 0.9982 |
| Kyn | 209.0920 | 2.79 | Y = -0.094633+0.0121749*X | 0.9988 |
| IA | 188.0706 | 4.75 | Y = -0.782103+0.0797002*X | 0.9972 |
| Trp | 205.0971 | 4.75 | Y = -0.169714+0.0208972*X | 0.9979 |
| Tryptamine | 161.1073 | 5.02 | Y = 0.00580657+0.00160669*X | 0.9989 |
| 5-HIAA | 192.0655 | 5.04 | Y = 0.0199783+0.00335059*X | 0.9972 |
| IAld | 146.0600 | 6.04 | Y = 0.100032+0.182304*X | 0.992 |
| IAA | 176.0706 | 6.19 | Y = 0.0148351+0.00249789*X | 0.9976 |
| Indole | 118.0651 | 6.04 | Y = 0.371255+0.0220199*X | 0.9978 |
| IAAld | 160.0756 | 6.53 | Y = -0.0291743+0.00368303*X | 0.9968 |
| IPA | 190.0862 | 6.6 | Y = -0.032452+0.00785679*X | 0.9986 |
| Trp-d5 |  |  | - |  |

m/z: mass-to-charge ratio; RT: retention time.
