# Supplementary material for: Effects of sulforaphane on breast cancer based on metabolome and microbiome
Source: Food Sci Nutr. 2023 Mar 31;11(5):2277–87. doi: 10.1002/fsn3.3168 (PMC10171519; doi:10.1002/fsn3.3168)
Supplement: Supplementary file 2 — Table S2 [file FSN3-11-2277-s001.docx]

**supplementary Table 2 MS instrumental parameters for one carbon metabolites**

| Metabolites | m/z | RT | Regression equation |  |  | R^2^ |  |  |
| --- | --- | --- | --- | --- | --- | --- | --- | --- |
| 5-MT | 460.1939 | 5.78 | Y = -0.00082576+0.000247474X |  |  | 0.9988 |  |  |
| Ser | 106.0498 | 0.89 | Y = -0.00154792+6.12984e-005X |  |  | 0.9994 |  |  |
| Gly | 76.0393 | 0.89 | Y = 0.000436178+1.03502e-005X |  |  | 0.9992 |  |  |
| Met | 150.0583 | 1.34 | Y = 0.169933+0.00115269X |  |  | 0.9913 |  |  |
| SAM | 399.1445 | 1.10 | Y = -0.00693517+0.0294037X |  |  | 0.9892 |  |  |
| SAH | 385.1289 | 3.75 | Y = -0.000414952+0.000269588X |  |  | 0.9999 |  |  |
| HCY | 136.0427 | 0.87 | Y= 0.007418+0.000102584X |  |  | 0.9979 |  |  |
| Betaine | 118.0862 | 0.91 | Y = 0.441816+0.042883X |  |  | 0.9949 |  |  |
| SAH-d4 | 389.1534 | 3.73 | - |  |  | - |  |  |
| MeT-d4 | 154.0831 | 1.34 | - |  |  | - |  |  |

m/z: mass-to-charge ratio; RT: retention time.
