# Supplementary material for: Effects of sulforaphane on breast cancer based on metabolome and microbiome
Source: Food Sci Nutr. 2023 Mar 31;11(5):2277–87. doi: 10.1002/fsn3.3168 (PMC10171519; doi:10.1002/fsn3.3168)
Supplement: Supplementary file 3 — Table S3 [file FSN3-11-2277-s002.docx]

**supplementary Table 3 The list of differential metabolites characterized after SFN intervention**

| No. | Metabolites | m/z(amu) | t_R_(min) | VIP score | Fold change  (SFN vs control) | P-value  (corrected) |
| --- | --- | --- | --- | --- | --- | --- |
| 1 | L-Methionine | 299.1092 | 7.03 | 4.04 | 0.17 | 0.0000 |
| 2 | Octadecanedioic acid | 337.2342 | 8.36 | 3.51 | 0.36 | 0.0000 |
| 3 | 3-Hydroxyhippuric acid | 196.0602 | 4.27 | 3.43 | 2.72 | 0.0000 |
| 4 | Uridine 5'-diphosphate | 368.9895 | 3.87 | 3.04 | 2.23 | 0.0001 |
| 5 | S-Lactoylglutathione | 380.1113 | 3.53 | 3.02 | 2.01 | 0.0001 |
| 6 | 1-Salicylate glucuronide | 337.0522 | 3.86 | 2.99 | 1.98 | 0.0001 |
| 7 | Glutamylalanine | 277.1049 | 7.16 | 2.99 | 3.76 | 0.0001 |
| 8 | DG (8:0/0:0/8:0) | 367.2445 | 8.65 | 2.87 | 0.07 | 0.0003 |
| 9 | Glycolic acid | 189.0016 | 5.63 | 2.84 | 0.23 | 0.0013 |
| 10 | 6-Methylmercaptopurine | 187.0059 | 5.61 | 2.83 | 0.32 | 0.0013 |
| 11 | 5,6-Dihydroxyindole | 150.0550 | 1.93 | 2.80 | 0.71 | 0.0004 |
| 12 | Palmitic acid | 535.4685 | 14.24 | 2.77 | 1.48 | 0.0006 |
| 13 | Adenosine monophosphate | 346.0548 | 2.04 | 2.76 | 0.60 | 0.0006 |
| 14 | Fructoseglycine | 218.0661 | 1.71 | 2.74 | 1.30 | 0.0006 |
| 15 | 3-Nitrotyrosine | 475.1067 | 0.79 | 2.73 | 0.68 | 0.0006 |
| 16 | Cer(d18:0/24:0) | 232.9847 | 5.56 | 2.72 | 0.25 | 0.0015 |
| 17 | alpha-Muricholic acid | 441.3189 | 9.56 | 2.72 | 0.56 | 0.0006 |
| 18 | Glutamylaspartic acid | 321.0938 | 5.61 | 2.72 | 2.43 | 0.0009 |
| 19 | S-(Formylmethyl)glutathione | 350.1006 | 2.75 | 2.67 | 1.63 | 0.0009 |
| 20 | 2-Methylguanosine | 320.0957 | 4.16 | 2.65 | 0.71 | 0.0014 |
| 21 | 6-Phosphogluconic acid | 335.0383 | 2.35 | 2.64 | 2.57 | 0.0011 |
| 22 | Guanosine monophosphate | 344.0396 | 1.96 | 2.52 | 1.24 | 0.0029 |
| 23 | 4-hydroxybenzoic acid-4-O-sulphate | 216.9804 | 3.27 | 2.40 | 1.28 | 0.0047 |
| 24 | 2-Hydroxyacetaminophen sulfate | 227.9962 | 5.04 | 2.40 | 0.64 | 0.0093 |
| 25 | Oxalic acid | 178.9828 | 4.50 | 2.35 | 1.35 | 0.0070 |
| 26 | N-Acetyl-L-aspartic acid | 373.0867 | 2.77 | 2.34 | 1.79 | 0.0069 |
| 27 | D-Glucose | 161.0444 | 3.46 | 2.30 | 1.34 | 0.0070 |
| 28 | 5-Formiminotetrahydrofolic acid | 248.0891 | 4.51 | 2.29 | 0.64 | 0.0099 |
| 29 | dTDP-D-glucose | 189.0323 | 5.54 | 2.28 | 0.73 | 0.0089 |
| 30 | 6-Methyladenine | 337.1037 | 8.44 | 2.28 | 0.37 | 0.0084 |
| 31 | Glycerylphosphorylethanolamine | 216.0624 | 5.54 | 2.20 | 1.51 | 0.0109 |
| 32 | Phenylacetylglycine | 174.0548 | 5.06 | 2.20 | 0.56 | 0.0143 |
| 33 | Kynurenic acid | 190.0491 | 10.99 | 2.16 | 0.75 | 0.0139 |
| 34 | N-Acetyllactosamine | 366.1393 | 4.47 | 2.11 | 1.46 | 0.0196 |
| 35 | L-beta-aspartyl-L-leucine | 227.1030 | 3.49 | 2.11 | 1.46 | 0.0175 |
| 36 | 13(S)-HPODE | 168.1127 | 1.37 | 2.11 | 1.83 | 0.0165 |
| 37 | Arachidic acid | 335.2928 | 9.99 | 2.09 | 0.81 | 0.0176 |
| 38 | Allochenodeoxycholic acid | 357.2817 | 8.16 | 2.07 | 0.60 | 0.0195 |
| 39 | MG (0:0/15:0/0:0) | 315.2533 | 8.64 | 2.06 | 0.59 | 0.0185 |
| 40 | 5-Hydroxytryptophol | 178.0856 | 6.49 | 2.04 | 1.42 | 0.0208 |
| 41 | Sulforaphane-N-acetylcysteine (SFN-NAC) | 341.0644 | 5.28 | 2.04 | 5.04 | 0.0248 |
| 42 | TG (13:0/a-15:0/10:0) | 356.2781 | 8.16 | 2.01 | 0.61 | 0.0255 |
| 43 | Indoxyl sulfate | 212.0017 | 2.45 | 2.01 | 1.51 | 0.0239 |
| 44 | L-Glutamine | 320.1623 | 4.59 | 2.00 | 0.65 | 0.0256 |
| 45 | Pantetheine | 162.0544 | 5.54 | 2.00 | 0.53 | 0.0303 |
| 46 | dCMP | 306.0483 | 1.71 | 1.98 | 1.49 | 0.0280 |
| 47 | DG (15:0/16:1(9Z)/0:0) | 553.4843 | 11.39 | 1.97 | 0.68 | 0.0260 |
| 48 | Cyclic GMP | 366.0220 | 1.95 | 1.97 | 1.15 | 0.0280 |
| 49 | PG (18:0/20:3(5Z,8Z,11Z)) | 401.2851 | 5.40 | 1.96 | 1.60 | 0.0345 |
| 50 | 1-Methylguanosine | 364.0874 | 4.37 | 1.96 | 0.66 | 0.0490 |
| 51 | 2-Hydroxybutyric acid | 231.0830 | 1.48 | 1.95 | 0.67 | 0.0277 |
| 52 | Tryptophyl-Alanine | 578.2792 | 5.56 | 1.95 | 1.33 | 0.0280 |
| 53 | 10-Oxooctadecanoic acid | 297.2428 | 9.11 | 1.93 | 0.83 | 0.0406 |
| 54 | 3-Methoxy-4-Hydroxyphenylglycol sulfate | 245.0118 | 5.69 | 1.92 | 4.64 | 0.0340 |
| 55 | Deoxycytidine | 286.1040 | 1.64 | 1.92 | 1.29 | 0.0346 |
| 56 | DG (18:2(9Z,12Z)/24:0/0:0) | 353.3228 | 9.98 | 1.91 | 0.82 | 0.0339 |
| 57 | Indoleacetic acid | 176.0701 | 6.28 | 1.91 | 0.82 | 0.0379 |
| 58 | 2'-deoxycytidine 3'-monophosphate | 340.0907 | 5.23 | 1.91 | 0.78 | 0.0350 |
| 59 | S-(2-Chloroacetyl) glutathione | 404.0284 | 1.34 | 1.90 | 1.20 | 0.0387 |
| 60 | Phosphoserine | 218.0416 | 3.73 | 1.90 | 1.73 | 0.0358 |
| 61 | TG (14:1(9Z)/14:1(9Z)/18:4(6Z,9Z,12Z,15Z)) | 406.2941 | 8.34 | 1.90 | 0.58 | 0.0340 |
| 62 | Norepinephrine sulfate | 248.0230 | 0.80 | 1.89 | 1.16 | 0.0428 |
| 63 | TG (14:0/14:0/18:4(6Z,9Z,12Z,15Z)) | 408.3097 | 8.63 | 1.86 | 0.60 | 0.0385 |
| 64 | Galactaric acid | 269.0528 | 2.35 | 1.85 | 1.23 | 0.0398 |
| 65 | Nicotinamide riboside | 531.1684 | 2.63 | 1.81 | 1.55 | 0.0445 |
| 66 | L-proline | 231.1335 | 4.29 | 1.80 | 1.62 | 0.0498 |
| 67 | Creatinine | 146.0920 | 1.08 | 1.80 | 1.21 | 0.0497 |
| 68 | 3-Indole carboxylic acid glucuronide | 318.0628 | 2.21 | 1.79 | 1.16 | 0.0497 |

m/z: mass-to-charge ratio; t_R_: retention time.
